# Supplementary material for: Survival Rates of Patients with Non-Small Cell Lung Cancer Depending on Lymph Node Metastasis: A Focus on Saliva
Source: Diagnostics (Basel). 2021 May 20;11(5):912. doi: 10.3390/diagnostics11050912 (PMC8161301; doi:10.3390/diagnostics11050912)
Supplement: Supplementary file 1 [file diagnostics-11-00912-s001.zip › diagnostics-1157030-supplementary.pdf]

diagnostics

MDPI

Article

Survival Rates of Patients with Non-Small Cell Lung Cancer Depending on Lymph Node Metastasis: A Focus on Saliva

Lyudmila V. Bel'skaya [1,*], Elena A. Sarf [1] and Victor K. Kosenok [2]

[1] Biochemistry Research Laboratory, Omsk State Pedagogical University, 644099 Omsk, Russia; nemcha@mail.ru
[2] Department of Oncology, Omsk State Medical University, 644099 Omsk, Russia; victorkosenok@gmail.com
* Correspondence: ludab2005@mail.ru and belskaya@omgpu.ru

Table S1. Characteristics of cohorts depending on the differences in the chemical composition of saliva in pN_0.

LNS	Variables	Favorable Prognosis	Unfavorable Prognosis
		**LDH**	
pN_0	LDH, U/L	>1294	<1294
	Age, years	61.8±1.93	60.3±1.86
	Gender	M—45, F—15	M—52, F—8
	pT	pT1—9, pT2—42, pT3—9	pT1—9, pT2—38, pT3—13
	Histological subtype	ADC—37, SCC—23	ADC—38. SCC—22
	Morphological growth forms	Peripheral—56, central—4	Peripheral—50, central—10
	Treatment type	Radical—37, combined—20	Radical—42, combined—16
	Smoking status	Smokers—27, non-smokers—33	Smokers—26, non-smokers—34
	Relapse status	Yes—13, no—47	Yes—20, no—40
		**DC**	
pN_0	DC, c.u.	<3.97	>3.97
	Age, years	59.7±1.95	62.1±1.82
	Gender	M—44, F—12	M—53, F—11
	pT	pT1—9, pT2—37, pT3—10	pT1—9, pT2—43, pT3—12
	Histological subtype	ADC—36, SCC—20	ADC—39, SCC—25
	Morphological growth forms	Peripheral—49, central—7	Peripheral—57, central—6
	Treatment type	Radical—35, combined—20	Radical—44, combined—16
	Smoking status	Smokers—21, non-smokers—35	Smokers—32, non-smokers—32
	Relapse status	Yes—13, no—43	Yes—20, no—44
		**LDH + DC**	
pN_0	LDH + DC	>1294, <3.97	<1294, >3.97
	Age, years	61.2±2.37	61.9±2.13
	Gender	M—23, F—7	M—31, F—3
	pT	pT1—5, pT2—19, pT3—6	pT1—5, pT2—20, pT3—9
	Histological subtype	ADC—17, SCC—13	ADC—19, SCC—15
	Morphological growth forms	Peripheral—27, central—3	Peripheral—29, central—5
	Treatment type	Radical—16, combined—13	Radical—23, combined—9
	Smoking status	Smokers—11, non-smokers—19	Smokers—16, non-smokers—18
	Relapse status	Yes—5, no—25 *	Yes—12, no—22

Note. *—differences are statistically significant, $p < 0.05$; ADC—adenocarcinoma, SCC—squamous cell carcinoma; M—male, F—female. LNS—lymph node status. LDH—lactate dehydrogenase, DC—diene conjugates.

Table S2. Characteristics of cohorts depending on the differences in the chemical composition of saliva in pN_1.

LNS	Variables	Favorable Prognosis	Unfavorable Prognosis
		**ICs**	
**pN_1**	ICs, mmol/L	<0.296	>0.296
	Age, years	59.8±2.49	60.4±3.31
	Gender	M—21, F—4	M—24, F—2
	pT	pT2—11, pT3—13, pT4—1	pT2—9, pT3—10, pT4—7
	Histological subtype	ADC—11, SCC –14	ADC –11, SCC—15
	Morphological growth forms	Peripheral—15, central—10	Peripheral—12, central—13
	Treatment type	Radical—3, combined—14, palliative—6	Radical—0, combined—13, palliative—13
	Smoking status	Smokers –12, non-smokers—13	Smokers—16, non-smokers—10
	Relapse status	Yes—16, no—9	Yes—16, no—10
		**MM**	
**pN_1**	MM, c.u.	>0.903	<0.903
	Age, years	60.4±2.77	59.8±3.11
	Gender	M—22, F—4	M—23, F—2
	pT	pT2—9, pT3—14, pT4—3	pT2—11, pT3—9, pT4—5
	Histological subtype	ADC—10, SCC—16	ADC—12, SCC—13
	Morphological growth forms	Peripheral—14, central—12	Peripheral—14, central—11
	Treatment type	Radical—2, combined—14, palliative—9	Radical—1, combined—13, palliative—10
	Smoking status	Smokers—11, non-smokers—15	Smokers—17, non-smokers—8
	Relapse status	Yes—17, no—9	Yes—15, no—10
		**ICs + MM**	
**pN_1**	ICs + MM	< 0.296, > 0.903	> 0.296, < 0.903
	Age, years	61.1±3.46	61.2±4.66
	Gender	M—13, F—2	M—15, F—0
	pT	pT2—5, pT3—9, pT4—1	pT2—5, pT3—5, pT4—5
	Histological subtype	ADC—6, SCC—9	ADC—7, SCC—8
	Morphological growth forms	Peripheral—9, central—6	Peripheral—8, central—7
	Treatment type	Radical—2, combined—8, palliative—4	Radical—0, combined—7, palliative—8
	Smoking status	Smokers—6, non-smokers—9	Smokers—11, non-smokers—4
	Relapse status	Yes—9, no—6	Yes—8, no—7

Note. ADC—adenocarcinoma, SCC—squamous cell carcinoma; M—male, F—female. LNS—lymph node status. ICs—imidazole compounds, MM—middle molecular toxins.

Table S3. Characteristics of cohorts depending on the differences in the chemical composition of saliva in pN_2.

LNS	Variables	Favorable prognosis	Unfavorable prognosis
		**Protein**	
**pN_2**	Protein, g/L	>0.583	<0.583
	Age, years	59.1±3.04	58.2±2.52
	Gender	M—27, F—6	M—27, F—7
	pT	pT3—16, pT4—17	pT3—20, pT4—14
	Histological subtype	ADC—16, SCC—17	ADC—17, SCC –17
	Morphological growth forms	Peripheral—16, central—16, mediastinal—1	Peripheral—16, central—16, mediastinal—2
	Treatment type	Radical—3, combined—9, palliative—18	Radical—2, combined—10, palliative—17
	Smoking status	Smokers—13, non-smokers—20	Smokers—17, non-smokers—17
	Relapse status	Yes—22, no—11	Yes—18, no—16
		**SB**	
**pN_2**	SB, c.u.	< 0.602	> 0.602
	Age, years	59.0±2.87	59.1±2.67
	Gender	M—27, F—7	M—28, F—6
	pT	pT3—21, pT4—13	pT3—15, pT4—19
	Histological subtype	ADC—18, SCC –16	ADC—12, SCC—13
	Morphological growth forms	Peripheral—16, central—16, mediastinal—2	Peripheral—17, central—16, mediastinal—1
	Treatment type	Radical—2, combined—10, palliative—19	Radical—3, combined—9, palliative—17
	Smoking status	Smokers—15, non-smokers—19	Smokers—16, non-smokers—18
	Relapse status	Yes—19, no—15	Yes—22, no—12
		**Protein + SB**	
**pN_2**	Protein + SB	> 0.583 + > 0.602; < 0.583 + < 0.602	< 0.583 + > 0.602; > 0.583 + < 0.602
	Age, years	58.6±2.59	59.3±4.66
	Gender	M—28, F—7	M—26, F—6
	pT	pT3—21, pT4—14	pT3—15, pT4—17
	Histological subtype	ADC—14, SCC—21	ADC—19, SCC—13
	Morphological growth forms	Peripheral—16, central—18, mediastinal—1	Peripheral—16, central—14, mediastinal—2
	Treatment type	Radical—3, combined—13, palliative—15	Radical—2, combined—6, palliative—20
	Smoking status	Smokers—14, non-smokers—21	Smokers—16, non-smokers—16
	Relapse status	Yes—21, no—14	Yes—19, no—13

Note. $p < 0.05$; ADC—adenocarcinoma, SCC—squamous cell carcinoma; M—male, F—female. LNS—lymph node status. SB—Schiff bases.
